# Associations of park access, park use and physical activity in parks with wellbeing in an Asian urban environment: a cross-sectional study

**DOI:** 10.1186/s12966-021-01147-2

**Published:** 2021-07-02

**Authors:** Nicholas A. Petrunoff, Ng Xian Yi, Borame Dickens, Angelia Sia, Joel Koo, Alex R. Cook, Wee Hwee Lin, Lu Ying, Ann W. Hsing, Rob M. van Dam, Falk Müller-Riemenschneider

**Affiliations:** 1 Saw Swee Hock School of Public Health, National University of Singapore and National University Health System, Block MD1, 12 Science Drive 2, #10-01, Singapore, 117549 Singapore; 2 Centre for Urban Greenery & Ecology, National Parks Board Singapore, 1E Cluny Rd., Singapore 11 Botanic Gardens, Singapore, 259569 Singapore; 3 Department of Psychological Medicine, Yong Loo Lin School of Medicine, National University of Singapore and National University Hospital System, 1E Kent Ridge Road, NUHS Tower Block, Level 9, Singapore, 119228 Singapore; 4 Department of Biomedical Data Sciences, Stanford Medicine, Stanford University, Palo Alto, California USA; 5 Department of Medicine, Stanford Prevention Research Center, Stanford School of Medicine, Stanford University, Palo Alto, California USA; 6 Digital Health Center , Berlin Institute of Health, Charite University Medical Centre Berlin, Kapelle-Ufer 2, 10117 Berlin, Germany

**Keywords:** Parks, Urban green space, Park access, Park use, Physical activity, Wellbeing

## Abstract

**Background:**

Relationships between park access, park use, and wellbeing remain poorly understood. The objectives of this study were to investigate: (1) perceived and objective park access in relation to park use and physical activity in parks; and; (2) perceived and objective park access, park use and physical activity in parks and their associations with wellbeing.

**Methods:**

An interviewer-assisted survey collected data on perceived time to walk to parks, park use time, park physical activity time and wellbeing (using a scale containing nine domains) amongst adult participants of the Singapore Multi-Ethnic Cohort. Geospatial maps of parks and the “walkable” street networks were created for the city-state of Singapore to objectively determine distances to accessible points on park boundaries. Multiple linear regression models estimated the importance of park access to park use and associations of park access and park use with wellbeing, adjusting for potential confounders.

**Results:**

Participants’ (*n* = 3435) average age was 48.8 years (SD, 12.8), 44.8% were male and 72.6% were of Chinese ethnicity. Better perceived but not true park access was significantly associated with greater park use. Park access (perceived or true) was not associated with physical activity time in parks. Greater participant park time and physical activity time in parks were associated with higher wellbeing scores (*p* < 0.001). The differences in wellbeing scores between the reference groups, who spent negligible time in parks, and the highest quartiles of time in parks (10.8 h/month) and physical activity in parks (8.3 h/month) were 3.2 (95% CI 2.1–4.4) and 4.2 (95% CI 4.1–6.3) points out of 100 respectively. These associations were similar for most domains of wellbeing, with clear dose-response relationships.

**Conclusions:**

While perceived park access was strongly associated with park use and well-being, true park access was not, and neither park access measure was associated with park physical activity. Future studies could investigate the influence of park attributes on park use, physical activity in parks and wellbeing. The consistent associations of park use and particularly physical activity in parks with wellbeing suggest that promoting park use, and especially physical activity in parks, is a promising strategy for improving wellbeing in urban settings.

**Supplementary Information:**

The online version contains supplementary material available at 10.1186/s12966-021-01147-2.

## Introduction

The built environment influences our health [1–3], and public parks and green spaces are particularly important to many aspects of our physical, social and mental health [4–9]. In cities, where increasingly most of the world’s population live [10], there is a need to study how habitants can benefit from green space. This is particularly salient in Asia where many of the world’s most populous cities are found [10], and since 90% of urban growth is expected to occur in Asia and Africa in the next 30 years [11].

The United Nations’ Sustainable Development Goals for 2030 include a goal for sustainable cities and communities, which cuts across many of the 17 goals [11]. In the Quito Declaration for Sustainable Cities, the United Nations detailed a ‘New Urban Agenda’ on sustainable cities and human settlements which included the statement, ‘We envisage cities and human settlements that prioritize inclusive, accessible, green and quality public spaces that are friendly for families, enhance social and intergenerational interactions … ’ [12]. The Singapore Green Plan for 2030 (Green Plan) released in 2021, states it was developed to strengthen Singapore’s response to United Nations sustainable development agenda [13]. The first of five pillars in the Green Plan is to become a ‘City in Nature’, and amongst several actions is one to plant a million more trees, and have every household within a 10-min walk from a park by 2030. This will almost double what is already a substantial amount of park land for the relatively small island-state. Therefore, Singapore provides an ideal opportunity to study relationships of park and greenspace access and use with the health and wellbeing of city-dwelling residents in Asia.

Conceptual models have described the relationships of parks and urban green space with health. The opportunities parks and green spaces provide for physical activity, social contact, stress reduction and associated benefits to physical and psychological health have been central to most of these [6, 8, 14–16]. The two most recently published models [8, 16] also reviewed key evidence in relation to each element. One found that there was consistent evidence for park and green space exposure being associated with reduced psychological stress, yet there were few studies that have investigated the associations of green space with social contact [8]. In regards to the relationships of exposure to green space with physical activity and health, one showed that there was inconsistent evidence for access to parks or green space being related to physical activity [8]. The other, which considered exposure to nature being associated with physical activity in depth, surmised that reviews of this literature generally find a positive, yet weak association [16]. The present study contributes to addressing research gaps on associations park and green space exposure with perceptions of social connectedness (a construct of wellbeing) and the poorly understood relationship of proximity to parks (a measure of park access) with physical activity in parks [17].

A recent systematic review on green space and mental wellbeing concluded that the evidence for relationships of access and use of green spaces with wellbeing is currently inconclusive and highlighted several research gaps [18]. In particular, they recommended that measures of wellbeing should combine multiple constructs since wellbeing is multi-faceted. In addition, studies to date have used different methods to measure access to green spaces which may have contributed to inconsistent results [19]. Complexities in measuring access to green spaces include the need to incorporate network analysis (e.g. walkable street networks instead of euclidian distance to a point within a certain radius “as the crow flies”), which considers the inclusion of truly accessible points in such network analysis (particularly for larger green spaces and parks since large sections may be inaccessible) and to evaluate the differences between perceived and objective access to these spaces. Whilst park and greenspace usage may seem like a relatively simple variable to measure, the evidence to date of a relationship with wellbeing has been hampered by a low volume of studies, inconsistent methods, and varying study quality [18].

The small number of studies that have assessed relationships between objectively measured park access, using distance via the street network, with park use or physical activity in parks have produced inconsistent findings [17, 20–24]. For example, a study using the distance to the geometric centroid of a park via the street network found no relationships with self-reported park visitation in the last 30 days, or with time spent being physically active in a park in a usual week [20]. In contrast, a study involving 458 participants found significant associations of objectively measured street network distance to a park via the accessible point on the park boundary and self-reported use of parks for participants living within 1.2 km [24]. In the latter study, the point on a parks boundary along the path on the street network was checked, and if this point was not accessible (e.g. a physical barrier such as a river or a fence) the access point was adapted. This may be particularly important for larger parks where, for example, accessing an entrance gate along a wide perimeter may add significant walking distance. The present study will apply this approach of considering distance to the nearest truly accessible point on the park boundary via the street network, but in a larger sample amongst all residential planning areas in the entire country of Singapore and combined with a measure of perceived time to walk to a park.

Several observational studies on the relationship between access and/or use of parks and green spaces with wellbeing have been conducted, including studies investigating relationships of the proportion of land allocated to parks and green space in an area with wellbeing [25–28], studies on perceived travel time to a park or green space and wellbeing [29–31], one study on objectively measured euclidian distance to a park or green space with wellbeing [29], and regular use of parks and other ‘natural environments’ for physical activity with wellbeing [29, 32]. Randomized-controlled trials of interventions aimed to promote physical activity in parks demonstrated a beneficial effect on quality of life [33] and reductions in stress [34]. However, we are not aware of studies of the relationship of green spaces including parks with different domains (i.e. individual constructs) of wellbeing or studies of relationships of parks with wellbeing that have been conducted in Asian cities.

To address these gaps, the objectives of our study were to investigate perceived and true park access and their associations with park use time and park physical activity time; and, associations of perceived and true park access, park use and park physical activity, with overall wellbeing and domains of wellbeing amongst a population-based cohort in Singapore, a city-state located in the Malay Archipelago that plans to be ‘a city in nature’ [13].

## Methods

### Study population and context

The Singapore Multi-Ethnic Cohort (MEC) is a prospective cohort study of environmental, lifestyle, and genetic determinants of non-communicable diseases, with regular, ongoing participant re-visits, comprising health screenings and interviewer-administered questionnaires [35, 36]. Detailed information about this study can be found on the study website [37]. Phase 2 of the MEC aimed to increase the total number of MEC participants, and initial recruitment of MEC phase 2 occurred between 2013 and 2016 through household visitations. Specifically, government housing estates in which over 80% of Singaporeans reside throughout all residential planning areas of the Urban Redevelopment Authority in Singapore were identified, and interviewers recruited participants by knocking on the door of apartments. Citizens and permanent residents of Singapore aged 21 to 75 years were eligible to participate in the cohort. The first follow-up of the MEC phase 2 cohort occurred from 2017 to 2021 and consisted of a home interview and a physical examination at a health screening centre. Between 1 December 2017 and 31 August 2019, the sample for this study was selected to be broadly representative of all MEC phase 2 participants using pragmatic methods. For example, since other studies involving surveys occurred during some of the time when the survey for the present study was being administered at the health screenings, every fifth attendee was invited to participate to avoid over-burdening participants. This study was conducted as part of the ‘Parks and Health’ project [38], which includes geospatial data analysis, a participant survey and an objective measurement cohort, to explore relationships of a comprehensive set of objective and perceived parks and green space exposure measures with health and wellbeing outcomes in existing cohorts [37]. This study used data from the geospatial analysis and the participant survey.

During the period questions on park access and park use were added to the existing MEC survey, 10,725 participants from the entire cohort attended health screenings. Of these, 7163 were not invited to do the survey since other surveys were occurring. Therefore, using pragmatic selection methods described above, 3524 participants were invited to complete the survey for this study. Of these, 88 participants were excluded for reasons including being unable to participate (because they were illiterate, unable to understand with assistance, or only spoke languages not supported by the research team), for instance Hokkien (*n* = 34); refused (*n* = 4); had missing demographic data (*n* = 6); or, indicated they spent more than 16 h in a park on a typical visit (*n* = 45), leaving a total of 3435 participants who were included in the final analysis. The ‘Parks and Health’ project was approved as part of an amendment to the Singapore Population Health Studies by the National University of Singapore Institutional Review Board, approval reference: B-16-125, 13–257.

### Measures

A web-based interviewer-assisted survey collected data from cohort participants on perceived park access, park use, usual modes of transport used to travel to parks, wellbeing, socio-demographic characteristics and physician-diagnosed chronic diseases during participant health screenings. To maximise the number of survey completions and the completeness of the survey data staff members ensured participants were able to access the survey and submit it when completed. The web-based version was available in English, English-Chinese and English-Malay. The Tamil translation was provided as a hard copy reference. The health screening also collected height and weight to calculate body mass index. To assess mode of transport to parks participants were asked how they mostly travelled to any park and back and they could select multiple options if their journey usually involved more than one form of transport per journey. The twelve response options were walking, running, cycling using a non-motorised bicycle, riding a non-motorised personal mobility device (e.g. scooter, skateboard), riding a motorised personal mobility device, by bus, by metro, by taxi, by ferry or boat, by car or truck, by motorbike or scooter and other. Measures which were included as exposures, outcomes and covariates are described in detail below.

### Park exposure variables

We adopted the National Parks Board of Singapore’s (NParks) definitions for public parks. Parks in this study were all public parks managed by NParks including smaller neighborhood or community parks (316), large regional parks (39) and nature reserves (11) [39]. This did not include public walking/cycling trails which are referred to as the Park Connector Network in Singapore [40]. After piloting, the description of parks below was placed in an introduction to the park-related questions, with names of example parks and photos to illustrate them:

***‘****The following questions are about your park usage and the activities you do in the park. When we ask about ‘parks’, we mean:*

Community parks which serve the immediate residents living in HDB* flats or private residential estates.

*Larger parks which include town parks, nature parks/reserves, coastal parks and offshore island.’*

*Housing Development Board (HDB) flats are publicly subsidised housing where over 80% of Singaporeans live [41]. They are generally large clusters of blocks which are densely populated.

Perceived park access was defined as the time in minutes (min) participants reported it takes to walk from their home to their nearest park in four categories of 1–5, 6–10, 11–20 and > 20 min. These categories correspond with a survey which has previously demonstrated strong reliability and validity [42], except the final two categories (21–30 and > 30 min) which were collapsed to > 20 min since negligible participants were in the category of > 30 min. To aid approximate comparisons between perceived park access and the true park access variable described below, categories were created based on studies of objectively measured walking times on street networks [43–46]. The categories were 0–399 m (1–5 min walk), 400–799 m (6–10 min walk), 800–1599 m (11–20 min walk) and > 1600 m (> 20 min walk).

The true park access is the objectively measured distance in meters on the “walkable street” network – mapped for the country of Singapore - surrounding each study participants’ home to the nearest accessible point on a park’s boundary. To create the “true” park access points, geospatial maps of all of Singapore’s public parks managed by NParks which were over 10000 m^2^ - since smaller parks are mostly accessible at any point and it is only a short distance to an accessible point for those that are not - were distributed to their Park Managers via an online survey tool in July 2019. The Park Managers printed their parks and marked accessible points before uploading them to the online survey tool. Research staff translated these into shape files in ArcGIS, checking the boundaries using Google maps and street view simultaneously. To enable calculations of distances to the nearest true park access point via the “walkable” street network, roads where pedestrians are prohibited such as freeways and toll roads were removed from the network. Using ArcGIS, the distance to the closest “true” park access point was then calculated by measuring the walkable distance from the residence to the nearest park’s accessible point based on the street network..

### Park use exposure and intermediate outcome variables

Park use time and park physical activity time were measured using two questions each, modelled on an established interviewer-administered physical activity questionnaire [47]. Park time was defined as the product of the number of days participants reported visiting a park in the last month and the time spent in a park on a typical visit. Similarly, park physical activity was the product of the number of days participants reported being physically active in a park or doing exercise in a park in the past month and the time spent engaging in physical activity in a park/exercising in a park on a typical day.

### Wellbeing outcome variables

As part of the Stanford WELL for Life Study, the WELL Singapore used the Stanford WELL for Life Scale (WELL), a multi-dimensional survey, to measure individual level wellbeing. The development of the WELL survey has been described elsewhere [48, 49]. Briefly, it used a grounded theory and qualitative research to identify domains of well-being in various cultural groups, including Asian (Chinese, Filipino, Japanese, Vietnamese) Americans, to create a tool for understanding wellbeing that is valid across cultures. Standard questions in each domain from internationally validated surveys were used to construct the survey of 53 questions in nine domains of wellbeing. A full list of the domains with definitions for each and the number of items per domain can be viewed in Additional file 1). The WELL survey and score were tested in four populations, including the San Francisco Bay Area in the US, New Taipei City in Taiwan, Hangzhou, China, and Singapore. The WELL survey asks respondents to rate their wellbeing for the past two to four-week time period. The nine domains of well-being included in WELL are: social connectedness, stress and resilience, experience of emotions, physical health, purpose and meaning, sense of self, financial security and satisfaction, spirituality and religiosity, and exploration and creativity. Each of the nine domains are scored 0–10, and an unweighted overall wellbeing score is calculated by summing each of the domain scores. For ease of interpretation, the score is re-scaled to 100. Although a 10th domain of lifestyle and daily practices has been included in previous studies [48], this domain was not included in this study since these are now considered determinants rather than components of wellbeing and will not be included in future studies using the WELL scale.

### Covariates

Covariates were selected based on the existing literature and knowledge of their potential to confound relationships of exposures with outcomes. Participants’ socio-demographic and anthropometric characteristics including age (years), gender (male, female), marital status (married, unmarried), household income (< 2000, 2000–3999, 4000–5999, > 6000 Singapore dollars per month), education level (None, Primary/Secondary, Post-secondary, University), ethnicity (Chinese, Indian, Malay and Other), and BMI (kg/m^2^) were considered as potential confounders. To enable calculations of BMI, weight was measured in light indoor clothes without shoes using calibrated digital scales with an accuracy of 0.1 kg. Body height was measured with the Frankfurt plane horizontal, to the nearest 0.1 mm without shoes using wall-mounted stadiometers. A chronic disease variable was created by combining self-reported physician-diagnoses of Type-II diabetes mellitus, heart attack, stroke, cancer and depression, defined from a yes/no response to the question, ‘Has a Western-trained doctor ever told you have each disease?’ Responses were used to generate the n (%) of participants who report being diagnosed. We also included smoking (smokers are defined as those who have smoked more than 100 cigarettes in their lifetime) and heavy alcohol consumers (defined as males who consume more than 5 servings of alcohol at a single drinking session in the past month, and females who consumed more than 4 servings) as covariates. A neighbourhood walkability index was created using geospatial data. For neighbourhood environmental characteristics, we included walkability as it is known to be associated with physical activity levels [50, 51] and possibly wellbeing [52]. Walkability was calculated with geospatial data using methods previously described by Frank and colleagues [53]. The geospatial data was used to create four components of the index - net residential density, retail floor area ratio, intersection density and land use mix entropy score. We used walkability scores within a walkable street network buffer of 500 m surrounding participants’ home address since it reflects the short distances Singaporeans walk overall during a day [46], and since evidence from a review suggests proximity of parks within smaller buffer zones is more likely to be associated with physical activity [54].

### Statistical analysis

Descriptive statistics summarised participant characteristics and the proportions and distributions of the exposures and outcomes amongst participants. All parks in Singapore were mapped geospatially across the country within planning areas, with the number of study participants per residential planning area colour-coded, and in relation to public housing where over 80% of Singaporeans live to observe how parks and study participants were spatially distributed using ArcGIS 10.8.1 (California, USA). Linear regression models were used to identify if environmental and behavioural exposures were associated with (1) park use and park physical activity of participants and (2) wellbeing as measured by WELL. Separate linear regression models for park use time per month and physical activity time in parks per month were fitted to perceived park access and true park access. We used unadjusted models (Model 1) and multivariable models with adjustments for socio-demographic characteristics (age, gender, ethnicity, marital status, work status, household income, education), BMI, presence of chronic diseases (diabetes, heart attack, stroke, cancer and depression), smoking, heavy alcohol consumption and the walkability index (Model 2). Models for wellbeing (based on an overall WELL score, and for each domain) were fitted to perceived park access, true park access, park use time per month and physical activity time in parks per month. All analyses were conducted using R version 3.6.1 (Vienna, Austria) [55]. Two-sided tests at the 5% level of significance were conducted, effect sizes and 95% confidence intervals are reported for the respective outcomes.

## Results

Table 1 shows the participant characteristics. The average age of participants was 48.8 years (SD, 12.8) and 44.8% were male. Participants were of Chinese (72.6%), Malay (13.4%), Indian (9.0%), and other (4.9%) ethnic groups. Monthly household income ranged from less than $2000 Singapore dollars (16.0%) to $6000 or above (33.2%) and 29% were university educated. Most (96.1%) participants resided in government housing estates. A combined 9.9% reported being diagnosed by a Western-trained physician with one of the specified chronic diseases. A total of 19.1% of participants were smokers and 9.1% reported heavy alcohol consumption. The average park use time was 10.6 h per month (SD 25.3 h/mo) and the average park physical activity time was 8.5 h/mo (SD, 21.9 h/mo) hours per month. On average, 76.0% of participants reported using a park in the past 30 days, and 63.0% reported they had used a park for physical activity in the past 30 days. Almost all (97.1%) participants reported mostly accessing parks by active modes of transport. The mean WELL score was 66.3 (SD 12.4) out of a possible 100.

**Table 1:** Participant characteristics

Characteristics	n (%)
Total	3435 (100)
Age, mean (SD)	48.8 (12.8)
Gender	
Male	1540 (44.8)
Ethnicity	
Chinese	2493 (72.6)
Malay	462 (13.4)
Indian	310 (9.0)
Others	170 (4.9)
Marital status	
Single (never married)	540 (15.7)
Married	2569 (74.8)
Divorced/widowed/separated	326 (9.5)
Work status	
Employed	2524 (73.5)
Household income (S$/month)	
Less than 2000	548 (16.0)
2000 to 3999	650 (18.9)
4000 to 5999	700 (20.4)
6000 and above	1141 (33.2)
Not reported	396 (11.5)
Education	
Secondary and below	1476 (43.0)
Pre-tertiary	963 (28.0)
University and above	996 (29.0)
Housing type	
Public housing estate	3302 (96.1)
Private condominium	88 (2.6)
Landed estate	3 (0.1)
Other	42 (1.2)
BMI, mean (SD)	24.86 (4.61)
Chronic disease^a^	340 (9.9)
Walkability^b^, mean (SD)	−0.24 (2.5)
Smokers^c^	638 (19.1)
Heavy alcohol consumers	307 (9.1)
Park user (in last 30 days)	2539 (76.0)
Park Use, hours/month, mean (SD)	10.63 (25.3)
Park physical activity (in last 30 days)	2104 (62.96%)
Park physical activity, hours/month, mean (SD)	8.52 (21.9)
Active mode of transport to parks ^c^	2820 (97.14%)
Inactive mode of transport to parks ^c^	83 (2.86)
Stanford WELL for Life Score, mean (SD)^d^	66.30 (12.4)
Domain 1: Social connectedness	6.60 (1.34)
Domain 3: Stress and resilience	6.22 (1.28)
Domain 4: Experience of emotions	6.26 (1.31)
Domain 5: Physical health	6.64 (1.45)
Domain 6: Purpose and meaning	6.76 (1.93)
Domain 7: Sense of self	7.01 (1.69)
Domain 8: Financial security and satisfaction	6.82 (2.71)
Domain 9: Spirituality and religiosity	6.57 (3.23)
Domain 10: Exploration and creativity	6.23 (2.29)

^a^Chronic disease variable generated by calculating the n (%) of participants who report being diagnosed by a Western-trained Medical Doctor with diabetes, heart attack, stroke, cancer and depression

^b^The mean of the overall normalised (using z-score) score. The sum of normalised scores for intersection density, net residential density retail floor area ratio and land-use mix, with the street connectivity weighted by two

^c^For transport to parks, active modes include walking, running, cycling, non-motorised personal mobility devices (e.g. scooter, skateboard), bus, train, ferry/boat (but not bus, train or ferry in combination exclusively with an inactive mode); inactive modes include car or truck, taxi, motorbike or scooter or motorised personal mobility devices (e.g. electric scooter) but not in combination with any of the aforementioned active modes

^d^Stanford WELL for Life total score is out of 100 and each Domain is out of 10

**Fig. 1:**
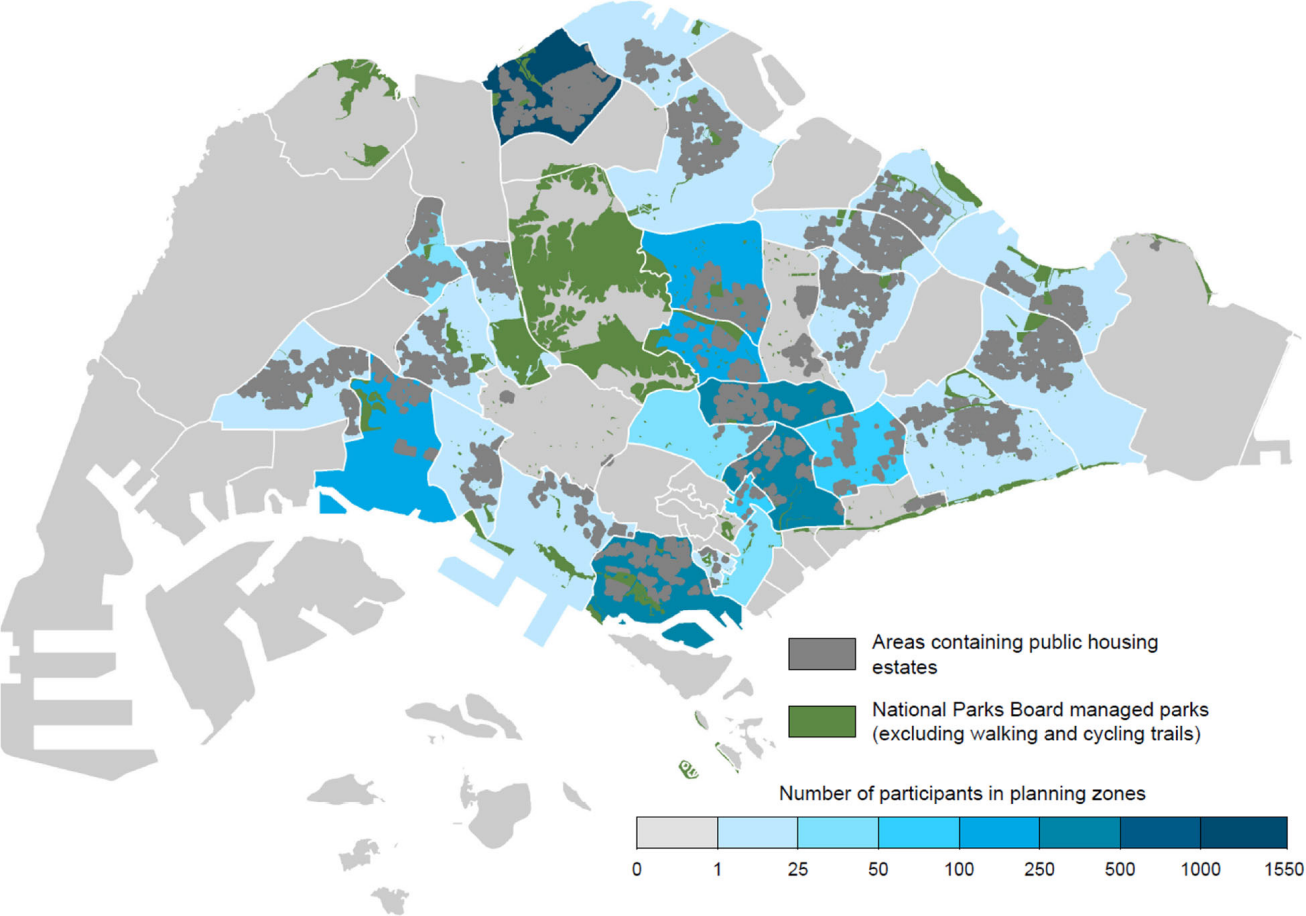
Spatial distribution of parks across and within planning areas across Singapore. Parks in green include larger regional parks, nature reserves and smaller community parks managed by the National Parks Board. Public housing estates where over 80% of Singaporeans live are dark grey and the white boundary lines represent the 55 planning areas of the Urban Redevelopment Authority. Light-grey non-residential areas are mostly industrial or wetlands and water catchments. Residential planning areas have been colour coded to show the numbers of participants within each

Figure 1 shows the spatial distribution of NParks managed public parks across Singapore by planning areas delineated by white outlines. Most parks are located among the planning areas in which public residences, shown in dark grey, are located. There is a large concentration of parkland centrally. We colour-coded the number of paticipants’ residing in each of the planning areas which are residential in blue tones, and included non-residential areas in light-grey. Paticipants’ were located in almost all of these residential planning areas, with reasonably even distribution of the number of participants per area, except for a concentration in one planning area in the north.

Table 2 summarises descriptive statistics for park access (true and perceived) and park use variables. Amongst participants, average walking time to their closest park was perceived to be less than 5 min (43.5%), 6 to 10 min (31.1%), 11 to 20 min (17.6%) and over 20 min (7.8%). True distance to their closest park was less than 400 m (11.5%), between 400 m and 799 m (27.9%), between 800 and 1599 m (34.5%) and 1600 m (26.1%) for participants. In our study the agreement between perceived and true park distance categories was poor (Spearman’s rank correlation rho = − 0.03, *p* < 0.07). Table 2 also presents unadjusted and adjusted associations of perceived and true park access with park use time and park physical activity time. Better perceived park access (i.e. less time to walk to a park), but not true park access was significantly associated with greater park use in both unadjusted and adjusted analyses. Both, perceived and true park access were not associated with park physical activity time.

**Table 2:** Levels of perceived park access and true park access and their associations with park use time and park physical activity time (hours/month)

	n (%)	Model 1 (unadjusted)	95% CI	Model 2 (adjusted^a^)	95% CI
**Park use time (hours/month)**					
Perceived park access (minutes walk)					
1–5 min	1453 (43.5)	Ref.	–	Ref.	–
6–10 min	1039 (31.1)	−2.50	−4.53 to − 0.46	−2.43	− 4.46 to − 0.39
11–20 min	589 (17.6)	−4.47	−6.92 to − 2.02	−4.45	−6.91 to − 1.99
> 20 min	261 (7.8)	− 4.88	−8.25 to − 1.51	−4.95	− 8.33 to − 1.57
Overall *p*-value		*< 0.001*		*< 0.001*	
True park access					
0–399 m	396 (11.5)	Ref.	–	Ref.	–
400–799 m	957 (27.9)	−1.59	−4.63 to 1.45	− 1.13	− 4.16 to 1.90
800–1599 m	1186 (34.5)	− 1.46	− 4.40 to 1.49	− 0.79	−3.87 to 2.29
> 1599 m	896 (26.1)	1.47	−1.57 to 4.55	2.20	− 0.96 to 5.37
Overall *p*-value		*0.034*		*0.027*	
**Park physical activity time (hours/month)**					
Perceived park access					
1–5 min	1453 (43.5)	Ref.	–	Ref.	–
6–10 min	1039 (31.1)	0.07	−1.70 to 1.83	0.10	− 1.66 to 1.86
11–20 min	589 (17.6)	−1.67	−3.79 to 0.45	− 1.69	− 3.82 to 0.43
> 20 min	261 (7.8)	−0.61	− 3.53 to 2.30	−0.73	− 3.65 to 2.18
Overall *p*-value		*0.416*		*0.386*	
True park access					
0–399 m	396 (11.5)	Ref.	–	Ref.	–
400–799 m	957 (27.9)	−2.67	−5.30 to −0.05	− 2.43	− 5.04 to 0.19
800–1599 m	1186 (34.5)	−2.84	− 5.38 to − 0.29	−2.62	−5.28 to 0.03
> 1599 m	896 (26.1)	−1.56	−4.20 to 1.09	− 1.09	−3.82 to 1.64
Overall *p*-value		*0.116*		*0.125*	

^a^Adjusted for age, gender, race, marital status, work status, household income, education, BMI, combined chronic diseases (heart attack, stroke, type II diabetes mellitus, depression and cancer), the walkability index within the walkable neighbourhood buffer of 500 m surrounding participants’ homes, smoking and alcohol consumption

Table 3 presents associations of perceived park access, true park access, park use time and park physical activity time with the WELL score. Better perceived but not true park access was significantly associated with higher total WELL scores in both unadjusted and adjusted analyses. In addition, park use time and park physical activity time were significantly associated with the total WELL score in both unadjusted and multivariable-adjusted analyses. Compared to the reference category each increase in park use time and park physical activity time were significantly associated with a corresponding increase in the overall WELL score in the unadjusted model and in the adjusted model, with a pattern reflecting a dose-response relationship.

**Table 3:** Associations of perceived park access, true park access, park use time and park physical activity (PA) time with WELL Scale wellbeing scores

	Model 1 (unadjusted)	95% CI	Model 2 (adjusted^a^)	95% CI
**WELL wellbeing scores**				
**Perceived park access**				
1-5 min	Ref.	–	Ref.	–
6-10 min	−1.01	−1.99 to − 0.03	−0.83	−1.77 to 0.11
11-20 min	−1.77	−2.95 to −0.58	− 1.49	− 2.62 to − 0.35
> 20 min	− 1.27	−2.90 to 0.35	−1.12	− 2.68 to 0.45
Overall *p*-value	*0.002*		*0.050*	
**True park access**				
0-399 m	Ref.	–	Ref.	–
400-799 m	−1.67	−3.14 to −0.21	− 1.08	−2.48 to 0.32
800-1599 m	−0.78	−2.20 to 0.64	− 0.50	− 1.92 to 0.93
> 1599 m	−1.74	− 3.21 to − 0.26	− 1.28	−2.74 to 0.19
Overall *p*-value	*0.046*		*0.215*	
**Park use time (hour/month)**				
1st quartile (0.00–0.02)	Ref.	–	Ref.	–
2nd quartile (0.03–3.04)	1.34	0.15 to 2.52	0.91	−0.23 to 2.05
3rd quartile (3.05–10.82)	4.08	2.90 to 5.25	3.14	2.00 to 4.28
4th quartile (> 10.82)	4.32	3.15 to 5.50	3.24	2.09 to 4.39
Overall *p*-value	< 0.001		< 0.001	
**Park PA time (hour/month)**				
1st quartile (0.00–0.07)	Ref.	–	Ref.	–
2nd quartile (0.08–2.07)	2.16	0.88 to 3.45	1.70	0.46 to 2.94
3rd quartile (2.08–8.32)	3.75	2.65 to 4.85	2.72	1.65 to 3.79
4th quartile (> 8.32)	5.20	4.14 to 6.26	4.16	3.12 to 5.19
Overall *p*-value	< 0.001		< 0.001	

^a^Adjusted for age, gender, race, marital status, work status, household income, education, BMI, combined chronic diseases (heart attack, stroke, type II diabetes mellitus, depression and cancer), the walkability index within the walkable neighbourhood buffer of 500 m surrounding participants’ homes, smoking and alcohol consumption

**Fig. 2:**
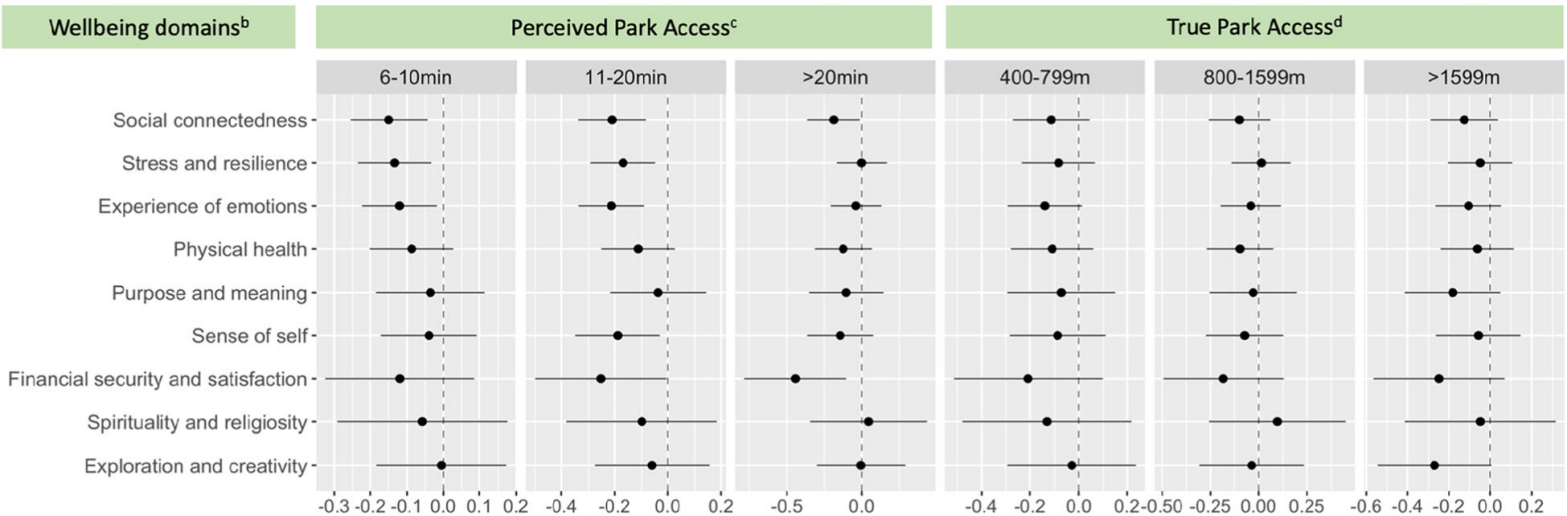
Adjusted^a^ associations of perceived park access and true park access with nine^b^ wellbeing scores from the WELL tool. ^a^Adjusted for age, gender, race, marital status, work status, household income, education, BMI, combined chronic diseases (heart attack, stroke, type II diabetes mellitus, depression and cancer), smoking and drinking status and the walkability index within the walkable neighbourhood buffer of 500 m surrounding participants’ homes. ^b^Associations reflect the magnitude of the change in the wellbeing score out of 10 in relation to changes in each park access and park use exposure. ^c^The reference group for perceived park access is 1-5 min. ^d^The reference group for true park access is 0 m – 399 m. The y-axis presents each of the nine domains of the WELL instrument. The x-axis shows the magnitude of the change in the wellbeing score out of 10 associated with each change in the level of perceived and true park access above the reference value. The dot in the middle is the average change in the WELL score associated with each change in the park access above the reference, whilst the ‘cats whiskers’ represent the 95% confidence intervals

**Fig. 3:**
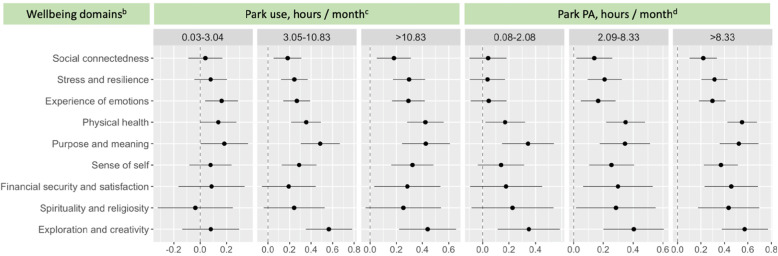
Adjusted^a^ associations of park use time and park physical activity (PA) time with wellbeing scores for nine^b^ domains of the WELL tool. ^a^Adjusted for age, gender, race, marital status, work status, household income, education, BMI, combined chronic diseases (heart attack, stroke, type II diabetes mellitus, depression and cancer), smoking and drinking status and the walkability index within the walkable neighbourhood buffer of 500 m surrounding participants’ homes. ^b^Associations reflect the magnitude of the change in the wellbeing score out of 10 in relation to changes in each park access and park use exposure. ^c^The reference group for perceived park us is 0.00–0.02 h/month. ^d^The reference group for true park access is 0.00–0.07 h/month. The y-axis presents the nine domains of the WELL instrument. The x-axis shows the magnitude of the change in the wellbeing score out of 10 associated with each change in the level of park use and park physical activity above the reference value. The dots in the middle are the average change in the WELL score associated with each change in the park use above the reference, whilst the ‘cats whiskers’ represent the 95% confidence intervals

Adjusted associations between park access, park use, and park physical activity with scores for different wellness domains are summarized in the forest plots in Figs. 2 and 3. The reference values for the levels of park access (Fig. 2) and park use (Fig. 3) are displayed in the legend. In Fig. 2, moving left to right across the rows for each domain, as the perceived distance to walk to a park was greater compared to the reference, which had a perceived walking distance of 0-399 m, there was a corresponding decrease in the wellbeing score out of 10 for five or the nine domains. In other words, participants who felt they lived further from a park reported significantly lower wellbeing scores for most domains of wellbeing. However, changes in the level of true park access were not significantly associated with changes in any of the WELL domain scores. In contrast, Fig. 3 shows that greater park use time was significantly associated with better WELL scores for most domains and park physical activity time was significantly associated with better scores for all WELL domains. The pattern displayed in the plots in Fig. 3 shows that as park use time and physical activity time in parks per month was higher from left to right across the rows, wellbeing scores for each domain also improved in comparison to the reference groups who reported almost no park use time or negligible physical activity time in parks per month.

## Discussion

This nationwide study in Singapore is unique as it used objective and subjective measures of park access as well as subjective measures of park use and physical activity in parks to evaluate their impact on wellbeing, assessed by a novel multi-dimensional WELL survey. Importantly, it contributes new research on parks and other green spaces as determinants of wellbeing in an urban Asian setting. We found greater park use and physical activity in parks were consistently associated with higher overall wellbeing scores as well as in most wellbeing domains, with clear dose-response relationships. Better perceived park access, but not true park access, was associated with greater park use and with better wellbeing. Neither perceived nor true park access was associated with physical activity in parks.

Our finding that perceived, but not true park access, was associated with park use warrants careful interpretation since studies investigating these associations among adults appear scarce and there is no consensus on appropriate measures. Consistent with other studies [24, 56, 57] our study found perceived access to parks was associated with park use, although there were differences in how each study operationalized the measures. A review suggested perceived distance to a park can be a useful measure supplementing objective distance measures [4], since it may be an important driver of park use where there is poor agreement between perceived and objective distance measures of park access. In our study the agreement between perceived and true park distance categories was poor, and many others report similar mismatches [23, 44, 45, 58, 59]. A study which included sub-group analysis with participants whose perceived and objective distance to a park agreed found that, amongst the sub-group, those living closer to parks were significantly more likely to engage in some physical activity in parks than those who perceived living further from a park. This led the authors to conclude that awareness of distance to parks may be a determinant of their use, and both park provision and promotion of their use may be important to their use for physical activity [23]. The only study we found which investigated associations of objective measures of park access with both park use and physical activity in parks also found that distance to a park via the walkable street network was not associated with park use or physical activity in parks [21]. Studies using other measures of park access, such as the number of parks, park area and features of parks located within a certain distance from participants’ homes have found relationships with park use or physical activity in parks [20–22]. Whilst the evidence of relationships between objectively measured park access and park use is inconsistent, park access is likely to be an important factor determining their use and perceived closer distance to a park does seem to be consistently associated with their use. Studies have suggested both perceived and objective park proximity measures be included [4, 23, 60], and one of these studies recommended similar perceived distance categories to those employed in our study be used in conjunction with an objective measure of the number of parks within one kilometer from residential addresses to increase comparability and validity of park access measures [60].

It seems plausible that the associations of park access with park use and with park-based physical activity are specific to contextual factors of the location including climate, topography, socio-economic conditions, neighbourhood design and transportation infrastructure. It is well established in the urban planning literature that the utilisation of public open space varies from context to context [61–63] and the importance of context has recently been empahsised by researchers with expertise in examining relationships of built and natural environments with physical activity [16]. An international study in 12 diverse cities across eight countries showed the distances to a park from participants’ residences via the walkable street network varied greatly between cities [60]. In our sample from Singapore, the majority (74%) lived within 1600 m of a park and about 40% lived within 800 m via the walkable street network. This is since Singapore has been planned along the lines of the ‘neighbourhood’ concept of British post-war new towns [64], and the neighbourhood park is centrally located within neighbourhoods of around 6000 dwelling units, whilst at the smaller precinct level about 0.2 ha of landscaped area with facilities for active and passive recreation are provided (e.g. children’s playgrounds, adult fitness corners and hard courts for ball games) to serve a few blocks comprising around 500 to 1000 dwelling units. Although the scales of outdoor space provided within neighbourhoods and precincts differs across cities and changes over time, this way of planning the provision of such outdoor space has been shown to be common to Asian cities including Kuala Lumpur in Malaysia and Delhi in India [61]. Therefore, in locations like Singapore where the distance to a park is reasonably close for the majority of the population, other measures such as access to different sized parks and green spaces [28], micro-scale features of parks [20], and scale of the planning level they are allocated at (e.g. broader regional, neighbourhood or precinct versus smaller cluster and block level) [61] may be more important determinants of park use and physical activity in parks to examine [16, 17].

Addressing our second objective by reporting on the relationships of park access and park use with most domains of wellbeing is unique in the literature. Conceptual models have hypothesized plausible relationships of parks and green space exposure with constructs of wellbeing such as stress, social connectedness and physical health [6, 8, 14], and reviews highlight few studies have quantified these relationships [5, 7, 18, 65]. Although less empirical evidence exists, there is also a plausible mechanism for the relationships we found of park use and physical activity in parks with the wellbeing domain of purpose and meaning, since the biophillia hypothesis asserts that human-beings’ search for a fulfilling existence and finding meaning in life is closely dependent on our relationship with nature [66], perhaps since we evolved in natural environments. Our study supports these hypothesized relationships by showing that better perceived park access, and greater park use and physical activity time in parks are all associated with improvements in WELL scores for the domains where plausible mechanisms for relationships with park exposure exist. In contrast, it is also evident that for the domains financial security and satisfaction as well as spirituality and religiosity - where a relationships with park use may not be expected - the relationships are generally weaker, less consistent and the confidence intervals are wider. Further, since we found consistent associations and dose-response relationships for park use with almost all of the nine domains of the WELL instrument and for physical activity in parks with all domains of the WELL instrument, promoting park use may be important to the overall wellbeing of residents in urban settings. Given hypothesized mechanisms for this relationship include an innate connection with nature since humans evolved in green spaces [6], it is possible that these relationships of park use with wellbeing are universal but further studies with diverse populations are required to demonstrate this.

We found that greater perceived but not true park access was associated with higher overall wellbeing. A study which tested associations of objective and subjective park and green space access with psychological distress in youth, found that whilst perceived travel time to parks and green space was associated with lower psychological distress, objectively measured Euclidian distance to the nearest park or green space was not [29]. Further, a study of adults from New York City found that lower perceived time to walk to a park from home was indirectly associated with fewer days of poor mental health via park-based physical activity in models assessing mediation effects, but only among those not concerned about park crime [30]. Contrary to our findings, a study in Los Angeles found associations of shorter objective distance from the closest urban parks to participants’ home address with decreased psychological distress [31]. The discrepant findings may also relate to distance to parks being a more important determinant of park use, and associated reductions in psychological distress, in the less compact city of Los Angeles. Since a recent systematic review of relationships of green space including parks with mental wellbeing found that the evidence of a relationship with park access is inconclusive [18] our study makes a valuable contribution to this research, yet more studies are required. In areas where access to parks in terms of distance from participants’ homes to a park is good for a high proportion of the population, it may be important to explore relationships of access to different sized parks or parks with different attributes rather than general park access with wellbeing.

Strengths of our study include examining associations with all of the domains of wellbeing within a comprehensive measurement tool, inclusion of geospatial data on objective park access to all public parks for the whole country of Singapore, a complementary subjective measure of perceived park access, and geospatially mapping truly accessible points on boundaries of parks over 10000 m^2^. However, our study also had several limitations. Firstly, the measure of park access was distance to the nearest park; we did not consider other measures of access such as size of parks or access to micro-scale features of parks as our analysis found most people live relatively close to parks. Secondly, despite providing participants with detailed instructions prior to interviewers asking park-related survey questions, misclassification of playgrounds and fitness corners for parks is a potential limitation in the study since most Singaporeans live in public housing with these kinds of facilities. Thirdly, the cross-sectional study design precludes us from making inferences about the sequence of cause and effect. The observation of dose-response relationships, on the other hand, adds confidence that the observed associations may be valid. Finally, determination of clinically meaningful differences or changes in WELL scores do not exist yet, although for scales of 0–100 it has been suggested that a difference of around five or about half a standard deviation is meaningful [67], and the differences in WELL scores between the groups with the highest and lowest levels of physical activity in parks approached this.

## Conclusions

This study contributes important evidence demonstrating strong associations between park use and physical activity in parks with overall wellbeing, as well as most domains of wellbeing, consistent with dose-response relationships. The association between the duration of physical activity in parks and wellbeing was particularly strong, and evident for all nine domains of wellbeing. Geospatial data demonstrated the majority of the population in Singapore lived reasonably close to a park on the walkable street network. In this context, whilst perceived park access was strongly associated with park use and wellbeing, true park access was not, and neither perceived nor true park access was associated with physical activity in parks. This warrants future studies to consider other park access measures such as access to parks with features reflecting park quality at the micro-scale, park size and park density within certain distances from people’s residences since they may be more important for park use and wellbeing in locations where most people live close to parks. Our findings suggest that promoting park use, and in particular physical activity in parks, is a promising strategy for improving wellbeing in urban settings.

## Supplementary Information

**Additional file 1.** Stanford WELL for Life Study Instrument. Lists domains, provides definitiions and lists number of items for each.

**Additional file 2.** STROBE checklist. Completed STROBE checklist for this study.

## Data Availability

The dataset(s) supporting the conclusions of this article are available upon reasonable request and after the National Parks Board of Singapore approves its provision.

## Abbreviations

PA: Physical activity
CI: Confidence interval
N, n: Number of participants
U.S.A.: United States of America
SD: Standard deviation
Kg: Kilogram
m^2^: Meters squared
BMI: Body mass index in kilograms per meter squared
WELL: Stanford Well for Life Scale
MEC: Singapore Multi-Ethnic Cohort
NParks: National Parks Board of Singapore
HDB: Housing Development Board publicly subsidized housing
min: Minute
GIS: Geographical information systems
