# Supplementary material for: Associations of park access, park use and physical activity in parks with wellbeing in an Asian urban environment: a cross-sectional study
Source: Int J Behav Nutr Phys Act. 2021 Jul 2;18:87. doi: 10.1186/s12966-021-01147-2 (PMC8254359; doi:10.1186/s12966-021-01147-2)
Supplement: Supplementary file 1 — Additional file 1. Stanford WELL for Life Study Instrument. Lists domains, provides definitiions and lists number of items for each. [file 12966_2021_1147_MOESM1_ESM.pdf]

Stanford WELL for Life instrument

WELL Domain	Definition	Number of items
Social Connectedness	Positive or negative relationships with others and how they influence well- being/wellness. Includes people who are family, friends, intimates and broader social network. Functions of social relationships include social support, social influence, social pressure, social undermining and social comparisons. Also includes how the characteristics and decisions of others impact one's own well-being or wellness (e.g., substance abuse of a loved one).	13
Stress and Resilience	Resilience is described as the ability to/experience of adapting to change or tendency to bounce back after illness or hardship, ability to effectively manage stress, ability to balance tasks. Stress is described as a feeling of overload, being overwhelmed, out of control, using the term "stress," inability to balance or manage tasks.	14
Experience of Emotions	Addresses different emotional states, including high arousal pleasant states (excitement, joy, exhilaration, enthusiasm), neutral pleasant states (happy, content, satisfied), low-arousal pleasant states (calm, secure, peaceful), high arousal unpleasant states (angry, afraid, anxious, frustrated, hurt), neutral unpleasant states (sad, uncomfortable), and low arousal unpleasant states (depressed, bored).	11
Physical Health	Physical symptoms including pain, physical fitness, energy level, ability to resist or fight off illness, and self-assessment of physical health.	4
Purpose and Meaning	May include anticipating/looking forward to the future, planning, sense of accomplishment, doing something valuable, reason for being on this earth, engagement, mention of motivation. Autonomy, lack of feeling constrained, sense of agency. Negative aspects of purpose/meaning can include feelings of failure, lack of accomplishment, a loss of purpose.	2
Sense of Self	Describes an understanding or questioning of one's own nature, capacity, or worth. Ability, confidence, knowledge, self-worth, self-satisfaction, and understanding of self.	5
Financial Security	Addresses financial security, money, income, wealth, self-sufficiency, financial benefits, monetary resources, or other material resources.	1
Spirituality and Religion	Anything having to do with connecting to the sacred or immaterial world (faith or religion) such as relationship with God, spiritual communities, religious activities or impact of spirituality on sense of self or life outlook. Practice of faith, outlook on life/resilience, sense of self, life course/evolution of beliefs, connection with others, personal relationship with God.	1
Exploration and Creativity	Someone being creative, being engaged in artistic activities, creative thinking, self-expression. Also includes new personal experiences such as travel or learning. Can include new hobbies as well as new life experiences and transformations, as well as change in perspective.	1

Source: Stanford WELL for Life Study team.
For information on the study: https://med.stanford.edu/wellforlife/research/stanford-well-for-life.html
Information on instrument: Heaney CA, Avery EC, Rich T, Ahuja NJ, Winter SJ. Stanford WELL for Life: learning what it means to be well. Am J Health Promot. 2017;31(5):449-50.
Relevant publication: Chrisinger BW, Gustafson JA, King AC, Winter SJ. Understanding where we are well: Neighborhood-level social and environmental correlates of well-being in the stanford well for life study. International journal of environmental research and public health. 2019;16(10):1786.
