# Supplementary material for: Associations of park access, park use and physical activity in parks with wellbeing in an Asian urban environment: a cross-sectional study
Source: Int J Behav Nutr Phys Act. 2021 Jul 2;18:87. doi: 10.1186/s12966-021-01147-2 (PMC8254359; doi:10.1186/s12966-021-01147-2)
Supplement: Supplementary file 2 — Additional file 2. STROBE checklist. Completed STROBE checklist for this study. [file 12966_2021_1147_MOESM2_ESM.pdf]

STROBE Statement—Checklist of items that should be included in reports of ***cross-sectional studies***

	Item No	Recommendation
**Title and abstract**	1	(*a*) Indicate the study's design with a commonly used term in the title or the abstract Done. Title page
		(*b*) Provide in the abstract an informative and balanced summary of what was done and what was found Done
**Introduction**		
Background/rationale	2	Explain the scientific background and rationale for the investigation being reported Done. Introduction Pages 3-4.
Objectives	3	State specific objectives, including any prespecified hypotheses Done. P4, line 88.
**Methods**		
Study design	4	Present key elements of study design early in the paper Done. Page 5, line 104 onwards.
Setting	5	Describe the setting, locations, and relevant dates, including periods of recruitment, exposure, follow-up, and data collection Done. Page 5, Study Population and Context
Participants	6	(*a*) Give the eligibility criteria, and the sources and methods of selection of participants Done. Line 105 page 5.
Variables	7	Clearly define all outcomes, exposures, predictors, potential confounders, and effect modifiers. Give diagnostic criteria, if applicable Done. Section 2.2, Measures pages 6-10.
Data sources/ measurement	8*	For each variable of interest, give sources of data and details of methods of assessment (measurement). Describe comparability of assessment methods if there is more than one group Done, pages 6-10.
Bias	9	Describe any efforts to address potential sources of bias. Done. Several, e.g. explaining definition of parks page 6 line 139, to reduce potential measurement bias of park use intermediate outcomes. Also lines 113-121, report on participant flow – very few invited not able to participate.
Study size	10	Explain how the study size was arrived at Done. Lines 113 3 528 invited- line 119 participated.
Quantitative variables	11	Explain how quantitative variables were handled in the analyses. If applicable, describe which groupings were chosen and why Done. Section 2.3 Statistical analysis, pages 10-11.
Statistical methods	12	(*a*) Describe all statistical methods, including those used to control for confounding Done - lines 252-257 under statistical analysis.
		(*b*) Describe any methods used to examine subgroups and interactions N/A
		(*c*) Explain how missing data were addressed. N/A
		(*d*) If applicable, describe analytical methods taking account of sampling strategy N/A
		(*e*) Describe any sensitivity analyses N/A
**Results**		
Participants	13*	(a) Report numbers of individuals at each stage of study—eg numbers potentially eligible, examined for eligibility, confirmed eligible, included in the study, completing follow-up, and analysed Done - Lines 113-119
		(b) Give reasons for non-participation at each stage Done - Lines 113-119
		(c) Consider use of a flow diagram N/A
Descriptive data	14*	(a) Give characteristics of study participants (eg demographic, clinical, social) and

		information on exposures and potential confounders Table 1
		(b) Indicate number of participants with missing data for each variable of interest
Outcome data	15*	Report numbers of outcome events or summary measures N/A
Main results	16	(*a*) Give unadjusted estimates and, if applicable, confounder-adjusted estimates and their precision (eg, 95% confidence interval). Make clear which confounders were adjusted for and why they were included Done – throughout results
		(*b*) Report category boundaries when continuous variables were categorized Done – thr
		(*c*) If relevant, consider translating estimates of relative risk into absolute risk for a meaningful time period N/A
Other analyses	17	Report other analyses done—eg analyses of subgroups and interactions, and sensitivity analyses. Correlation co-efficient mentioned bottom column1 p6
**Discussion**		
Key results	18	Summarise key results with reference to study objectives Done
Limitations	19	Discuss limitations of the study, taking into account sources of potential bias or imprecision. Discuss both direction and magnitude of any potential bias. Done. Lines 445-457.
Interpretation	20	Give a cautious overall interpretation of results considering objectives, limitations, multiplicity of analyses, results from similar studies, and other relevant evidence Done
Generalisability	21	Discuss the generalisability (external validity) of the study results. Done – for example lines 418-20, finding may be generalizable.
**Other information**		
Funding	22	Give the source of funding and the role of the funders for the present study and, if applicable, for the original study on which the present article is based Done in declarations section.

*Give information separately for exposed and unexposed groups.

Note: An Explanation and Elaboration article discusses each checklist item and gives methodological background and published examples of transparent reporting. The STROBE checklist is best used in conjunction with this article (freely available on the Web sites of PLoS Medicine at http://www.plosmedicine.org/, Annals of Internal Medicine at http://www.annals.org/, and Epidemiology at http://www.epidem.com/). Information on the STROBE Initiative is available at www.strobe-statement.org.
